# Ultrastretchable Fiber Sensor with High Sensitivity in Whole Workable Range for Wearable Electronics and Implantable Medicine

**DOI:** 10.1002/advs.201800558

**Published:** 2018-07-23

**Authors:** Lianhui Li, Hongyi Xiang, Yan Xiong, Hui Zhao, Yuanyuan Bai, Shuqi Wang, Fuqin Sun, Mingming Hao, Lin Liu, Tie Li, Zhenhuan Peng, Jiaqiang Xu, Ting Zhang

**Affiliations:** ^1^ School of Nano Technology and Nano Bionics University of Science and Technology of China 96 Jinzhai Road Hefei Anhui 230026 P. R. China; ^2^ i‐Lab Suzhou Institute of Nano‐Tech and Nano‐Bionics (SINANO) Chinese Academy of Sciences (CAS) 398 Ruoshui Road Suzhou 215123 P. R. China; ^3^ Key Laboratory of Vehicle Crash/Bio‐Impact and Traffic Safety, Department 4th Institute of Surgery Research Daping Hospital Chongqing 400042 China; ^4^ Department of Chemistry Shanghai University Shanghai 200444 China

**Keywords:** fibers, implantable devices, strain sensors, ultrastretchable materials, wearable sensors

## Abstract

Fast progress in material science has led to the development of flexible and stretchable wearable sensing electronics. However, mechanical mismatches between the devices and soft human tissue usually impact the sensing performance. An effective way to solve this problem is to develop mechanically superelastic and compatible sensors that have high sensitivity in whole workable strain range. Here, a buckled sheath–core fiber‐based ultrastretchable sensor with enormous stain gauge enhancement is reported. Owing to its unique sheath and buckled microstructure on a multilayered carbon nanotube/thermal plastic elastomer composite, the fiber strain sensor has a large workable strain range (>1135%), fast response time (≈16 ms), high sensitivity (GF of 21.3 at 0–150%, and 34.22 at 200–1135%), and repeatability and stability (20 000 cycles load/unload test). These features endow the sensor with a strong ability to monitor both subtle and large muscle motions of the human body. Moreover, attaching the sensor to a rat tendon as an implantable device allowes quantitative evaluation of tendon injury rehabilitation.

In recent years, fast progress in material science has led to the development of flexible and stretchable electronics.^1^, ^2^, ^3^ In general, three configurations can be employed to produce stretchable electronics: i) rigid functional device islands and stretchable interconnects; ii) intrinsically stretchable functional device components; and iii) a combination of (i) and (ii).^4^ Although conventional metals and silicon have a certain degree of deformability by combining with the various stretchable structural designs, such as buckling,^5^ a wavy shapes,^6^ and a serpentine architecture,^7^ these materials cannot withstand dramatic mechanical deformation. Moreover, when stretchable electronic devices are mounted on human skin or curved surfaces, the mechanical mismatches between the devices and soft human tissue will lead to response failure. Therefore, mechanically superelastic and compatible sensors for wearable and implantable electronics are urgently required.

Typically, the sensing materials include metal nanowires,^8^, ^9^, ^10^ conducting polymers,^11^ and carbon nanomaterials, such as carbon nanotubes^12^, ^13^ and graphene,^14^, ^15^ and so on. However, currently, very few stretchable strain sensors based on these materials simultaneously possess a large workable strain range and high sensitivity, which severely limits their applications. For example, an elastic wearable carbon nanotube fiber strain sensor can be maximally stretched over 900%; however, over a 0–400% strain range, and the gauge factor (GF) was only 0.54.^16^ On the contrary, a graphite‐based strain sensor achieved a high GF of 536.6, which was mainly attributed to the generated cracks and overlaps of contacting areas between graphite‐slices. However, this high sensitivity was only found over a limited strain range of −0.62% to +0.62%.^17^ Development of highly sensitive and ultrastretchable strain sensors that have a large workable strain range is still difficult.

Buckled structures are usually used to fabricate stretchable conductors with stable conductance during deformation, in which conducting thin layers are deposited on the surface of a prestretched substrate followed by stretch‐release.^18^, ^19^, ^20^ Here, we report a novel buckled sheath–core fiber‐based ultrastretchable strain sensor and demonstrate its outstanding sensing performance in whole workable range. The ultrastretchable fiber sensor with a buckled sheath was designed and fabricated by wrapping an ultralight multiwalled carbon nanotubes/thermal plastic elastomer (MWCNT/TPE) composite film (NTTF) around a prestretched TPE elastic rubber fiber and then releasing the stretching force,^18^, ^21^, ^22^which is denoted as NTTF*_n_*@fiber, where *n* indicates the number of NTTF layers of the sheath. The 1D fiber strain sensor has a large workable strain range (>1135%), compatible elastic modulus with human skin (≈140 kPa), fast response time (≈16 ms), high sensitivity in whole workable range (GF of 21.3 over a 0–150% strain range and 34.22 over a 200–1135% strain range), and repeatability and stability (20 000 cycles load/unload test). We also demonstrated the performance of the sensor for monitoring both subtle muscle motions (such as arm muscles motion, drinking) and large motions (such as joint movements) of the human body. Furthermore, by attaching the sensor as an implantable device on the tendon of a lab rat, we were able to quantitatively evaluate tendon injuries.

**Figure** **1**a illustrates the fabrication procedure for the ultrastretchable sensitive sheath–core fiber sensors. Uniform NTTF was prepared on silica glass using a spray‐coating method. To enhance its conductivity, the film was immersed in absolute ethanol to partially dissolve the TPE and expose MWCNTs on the surface.^23^ Then, the NTTF was wrapped around a prestretched TPE fiber core. After releasing the prestretched TPE core, periodic bucklings formed along the fiber axial direction (see the inset of Figure 1a,b) and a wrinkled NTTF sheath–core fiber strain sensor was obtained. Both the superelastic TPE core and buckled microstructure of the composite sheath endowed the fiber strain sensor with large stretchability (Figure 1c).

**Figure 1:**
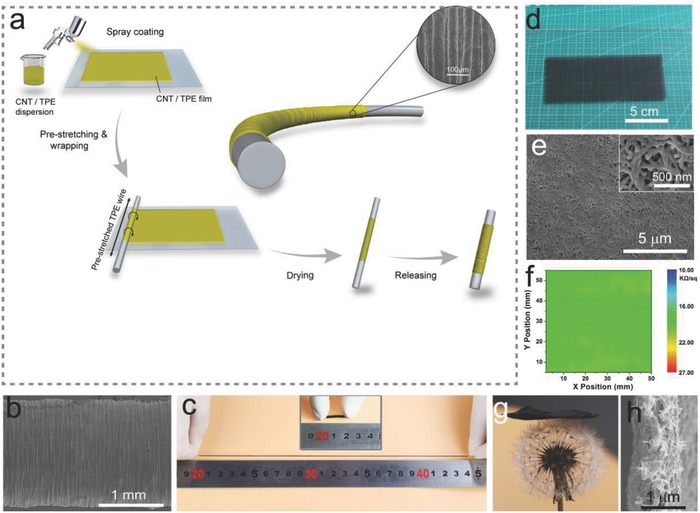
Preparation and characterization of the ultrastretchable fiber strain sensor. a) Schematic illustration of the fabrication procedure for the strain sensor. b) SEM image of the NTTF*_n_*@fiber strain sensor with five layers of NTTFs. The fabrication of the strain sensor was 1600%. c) Optical images showing a fiber strain sensor at a 1100% strained state and relaxed state (Inset), respectively. d) Photograph of the MWCNT/TPE composite film (NTTF) with a filler load of 12 wt% on silica glass. The size of the film was 15 × 8 cm^2^. e) SEM image of a 12 wt% MWCNT/TPE composite film treated with ethanol for 2 min. The inset shows a high‐resolution SEM image. f) Sheet resistance distribution of a 12 wt% MWCNT/TPE composite film measured over an area of 50 × 50 mm^2^. g) Photograph of a MWCNT/TPE composite film (2.5 × 2.5 cm^2^) on a dandelion. h) SEM image showing the cross‐sectional micromorphology of a MWCNT/TPE composite film treated with ethanol for 2 min. The thickness of the film was 800 nm.

As the sensing component of the wrinkled NTTF sheath–core fiber strain sensor, the NTTF sheath needed to have appropriate electrical and mechanical properties. Since the MWCNT content significantly affects the properties of the NTTF sheath, NTTF films with different MWCNT contents were prepared, and the mechanical and electrical properties were investigated to determine the optimal MWCNT content. The stress–strain curves are shown in Figure S1a (Supporting Information), and the calculated elastic modulus and maximum strain (ε_max,_ where the NTTF film breaks) are shown in Figure S1b (Supporting Information). As MWCNTs (elastic modulus ≈50 GPa)^24^ were introduced into the soft TPE matrix (elastic modulus ≈100 kPa) and the MWCNTs content increased from 8% to 16%, the elastic modulus of the composite increased from 8 to 58 MPa, while ε_max_ decreased from 626.29% to 90.5%. The sheet resistance of the NTTF decreased from 41.8 to 13.42 kΩ sq^−1^ as the weight percentage of the MWCNTs increased (Figure S1c, Supporting Information) because the higher concentration of MWCNTs formed a denser electrical conductive path (Figure S2a–e, Supporting Information) according to the percolation theory.^25^, ^26^ Figure 1d shows an optical photograph of the 12 wt% NTTF (length: 150 mm, width: 10 mm, thickness: 800 nm) on a glass substrate. Both the photograph and scanning electron microscopy (SEM) image (Figure 1e) indicate that the film is uniform without apparent agglomeration. A higher magnification SEM image (the inset of Figure 1e) shows that the composite film is formed from an interconnected network with MWCNTs as the backbone and TPE coating layers. The representative sheet resistance distribution of a 50 × 50 mm^2^, 12 wt% NTTF was measured as 18.995 ± 1.325 kΩ sq^−1^, with a deviation of less than 7% (Figure 1f), and this mesh structure endowed the nanocomposite with light and soft features (Figure 1g, the thickness was 800 nm; Figure 1h). By comprehensively considering of the conductivity, stretchability, and uniformity of the NTTF, 12 wt% MWCNTs was selected as the optimum filler loading for sensitive sheath layers.

Furthermore, NTTF*_n_*@fiber sensors with various fabrication prestrains (ε_pre_) were investigated since ε_pre_ of the TPE core directly affects the buckling of the sheath. The strain sensing performance was evaluated by measuring the relative resistance change (Δ*R*/*R*_0_) of the sensors, where Δ*R* = *R*–*R*_0_, and *R*_0_ and *R* are the resistances of the fiber sensors at relaxed and stretched states, respectively. For sensors with ε_pre_ = 0, the approximated linear slopes of the relative resistance change were 5.366 in 0–50%, 0.742 in 50–275%, and 387 in 275–350% (**Figure** **2**a). Here, the slope of the curve represents the gauge factor *GF* = (Δ*R*/*R*_0_)/ε, which represents the sensitivity to strain, where ε is the strain of the sensors. As shown in Figure 2a‐right1, a small strain (under 50%) decreased the contact area between the MWCNTs filled in a NTTF.^27^ As the strain increased from 50% to 275%, periodic bucklings perpendicular to the fiber axis gradually formed due to the reduction of the fiber diameter according to the Poisson effect, and the adjacent buckles began to contact one another (Figure 2a‐right2). Therefore, the current was able to flow through the contact point, which increased the axial electrical path and compromised the resistance increase caused by axial stretching. Under excessive strain (275–350%, Figure 2a‐right3), the NTTF sheath cracked and the resistance drastically increased.

**Figure 2:**
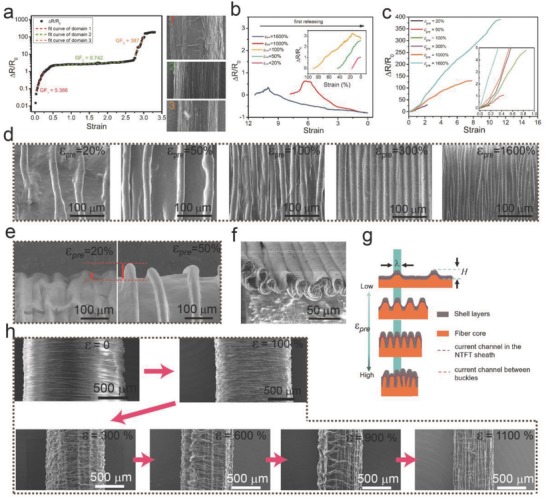
Performance and working mechanism characterization of the NTTF*_n_*@fiber sensors. TPE cores (diameter: 2 mm) were prestretched to 0%, 20%, 50%, 100%, 300%, 1000%, and 1600% strain. Five layers (*n* = 5) of NTTF (The single‐layer thickness is 800 nm) were wrapped as the sheath and then released to obtain sheath–core fiber strain sensors with different ε_pre_. a) Relative resistance change as a function of the tensile strain and linear fittings for a NTTF_5_@fiber sensor without prestrain (ε_pre_ = 0) in fabrication. SEM images with different number on the right show the surface morphology of the NTTF_5_@fiber sensor under different strain sensors. (1, 2, and 3 for strain = 0%, 200%, and 300%). b) Change of the resistance of NTTF_5_@fiber sensors with different ε_pre_ during the first release process. c) Resistance change as a function of strain for NTTF_5_@fibers with different ε_pre_. d) SEM images of NTTF_5_@fiber sensors with different ε_pre_ in the relaxed state. e) SEM images of the fiber sensor edge showing the height change of the buckles. f) Cross‐sectional image of the buckles of NTTF_5_@fiber sensors at 0% strain. The fabrication strain was 500%. g) Schematic illustration of the section morphology and current path of NTTF_5_@fiber sensors with different fabrication ε_pre_. h) SEM images of the surface morphology of the NTTF_5_@fiber sensor with different strain (ε). Fabrication of the strain sensor was 1600%.

For sensors with different ε_pre_ (ε_pre_ > 0) values, resistance changes during the first release in fabrication and subsequent stretching process needed to be investigated because the sheath morphology and conductive network in the NTTF sheath significantly changed in these processes. As shown in Figure 2b, the resistances of the sensors with 20% and 50% ε_pre_ were always increasing, while the resistances of the sensors with more than 50% ε_pre_ tended to increase first and then decrease during the first release. In particular, the final (after release) resistance was smaller than the initial resistance (after wrapping the NTTF sheath without release). For example, only one‐tenth of the initial resistance was observed for sensors with 1000% ε_pre_. The resistance change of sensors with different ε_pre_ values during stretching was then investigated. As shown in Figure 2c, the maximum strain (ε_max_) range increased from 18% to 1135% as ε_pre_ increased from 20% to 1600%, and a 1135% strain range was achieved when ε_pre_ was 1600% (Figure S3, Supporting Information). ε_max_ is defined as (*L*_max_ – *L*_0_)/*L*_0_, where *L*_max_ is the fabrication length of the NTTF sheath and *L*_0_ is the length of the released strain sensor. Note the experiment was set to stop when the length (*L*) of the sensor reached *L*_max_, which is the maximum point that can ensure a stable and reliable performance of the NTTF*_n_*@fiber sensor. The sensitivity of the sensors also showed a similar tendency to the ε_max_ range (Figure S4a–c, Supporting Information). The resistance change (Δ*R*/*R*_0_) of a NTTF_5_@fiber sensor with ε_pre_ = 20% was only 0.2, and the GF value was 1.35 over the whole strain range from 0% to 17%. However, the Δ*R*/*R*_0_ of a NTTF_5_@fiber sensor with ε_pre_ = 1600% changed to be as large as 390 for ε = 1135%, and the GF was 21.3 for a strain range from 0% to 150% and 34.22 for a strain range from 200% to 1135%. The sensitivities over the whole strain range were better compared to those of previously reported highly stretchable strain sensors.^28^, ^29^, ^30^, ^31^, ^32^

The formation of a buckled microstructure on the sheath surface plays an important role in understanding the sensing mechanism and high performance of NTTF*_n_*@fiber sensors. Figure 2d shows that axial buckles gradually formed along the fiber, approached one another, contacted each other, and eventually overlapped as ε_pre_ increased. Interestingly, there was no significant change in the peak width (λ) of the buckles and only the peak height (*H*) increased as ε_pre_ increased (Figure 2e), was mainly due to the minimization of the total elastic energy in the thin layer and soft substrate.^33^ From the buckling microstructure change, the mechanism of sensing performance with different ε_pre_ can be described as follows. In the process of the first release, the NTTF sheath suffers axial compression stress and tensile stress perpendicular to the fiber core axis, both of which are caused by the contraction of the fiber core.^34^ For sensors with a small prestrain (ε_pre_ ≤ 50%), tensile stress perpendicular to the fiber core axis was the primary cause of the contraction of the fiber core, which decreased the contact area between the MWCNTs filled in the NTTF, resulting in increased resistance (the resistance in the NTTF sheath is denoted as *R*_in_). At this point, the buckles do not contact each other and cannot appreciably change the conduction path, therefore; the sensitivity is only 1.35 for sensors with ε_pre_ = 20%. However, when ε_pre_ is large enough (ε_pre_ ≥ 100%), the buckles come into contact and the contact area between the adjacent buckles of the NTTF sheath increases as ε_pre_ increases (Figure 2f). The contact significantly affects the current pathway of the NTTF sheath due to the formation of an additional contact pathway between buckles (the contact resistance is denoted as *R*_contact_, Figure 2g). The current pathway between the buckles and conduction pathway in the NTTF sheath acts as two resistors in parallel, as shown in the resistor network model in Figure S5 (Supporting Information). According to the parallel circuit, the relationship between *R*_in_, *R*_contact_ and total resistance *R* can be described by Equation (1)

(1) $$1/R=1/R_{\mathrm{in}}+1/R_{\mathrm{contact}},\;1/R_{\mathrm{contact}}\propto A$$

where *A* is the average interbuckle contact area in the direction perpendicular to the fiber axis. A larger ε_pre_ not only leads to a larger strain range but also increases the contact area, which effectively reduces *R*_contact_. Apparently, the final resistance is only one‐tenth of the initial state, indicating that prestrain induced *R*_contact_ is the dominant factor for the for NTTF_5_@fiber sensors during the first release. Hence, the resistance of the NTTF_5_@fiber sensors increases first due to the tensile stress perpendicular to the fiber core axis and then decreases during the first release. In the stretching process, the buckles separate gradually (as shown in Figure 2h) and the contact area between the buckles decreases, which leads to a sharply increased *R*_contact_. The strain‐gauge‐enhancement configuration enables the contact areas between the buckles to decrease as the strain increases over a large range, resulting in a large resistance change. Therefore, the prestrain‐induced buckled microstructures perpendicular to the fiber core axis improves the strain range and sensitivity of the NTTF*_n_*@fiber sensors.

The essence of the buckles is the relocation of a new equilibrium state due to the mismatch of the two equilibrium states between the soft elastomeric TPE fiber core substrate and hard NTTF sheath surface layer, which occurs when the substrate spring force exceeds the critical threshold for buckling of the surface thin layer.^33^ Except for ε_pre_, changing the thickness of the sheath by controlling the thickness of a single NTTF layer (noted as *h*_s_) or then numbers of the NTTF layers of the sheath (*n*) can also affect the buckling morphology and sensing performance. **Figure** **3**a shows that NTTF_5_@fiber sensors (ε_pre_ is 500%) with thinner *h*_s_ exhibited a larger strain range (194%, 278%, 316%, 400%, and 426% strain for *h*_s_ = 2.4, 1.2, 1, 0.8, and 0.6 µm, respectively; the method to control the thickness of NTTF is introduced in the Experimental Section). The sensitivities of the NTTF_5_@fiber sensors with different *h*_s_ were demonstrated by piecewise fitting of the relative resistance changes versus the strain curve, as shown in Figure 3b. SEM images show that λ of the buckles increased from 13.3 to 44 µm as *h*_s_ increased from 0.6 to 2.4 µm (Figure 3c–g; and Figure S8, Supporting Information). These results can be attributed to the fact that the peak width (λ) and peak height (*H*) are strongly related to the thickness of the sheath layer (*h*), which can be theoretically described as

(2) $$H=h\sqrt{\frac{\varepsilon_{\mathrm{pre}}}{\varepsilon_{c}}-1},\;\lambda =\frac{\pi h}{\varepsilon_{c}},\;h=nh_{s}$$

where ε_c_ is the critical strain for wrinkles and *n* is the number of NTTF layers of the sheath. Equation (2) illustrates that *H* and λ are proportional to the thickness of the shell composites layer (*h*).^35^λ decreases with the decrease of *h*_s_, resulting in an increased lateral contact area between adjacent buckles and a larger resistance change (shown in Figure 3h). According to the definition of sensitivity (*GF* = (Δ*R*/*R*_0_)/ε), when *h*_s_ ≥ 0.8 µm, the influence of *R*_0_ is relatively small compared to the resistance change and sensors with a thinner NTTF have a higher Δ*R* and greater sensitivity. However, when *h*_s_ < 0.8 µm, *R*_0_ increases sharply (such as *h*_s_ = 0.8 µm, *R*_0_ ≈40 kΩ, and *h*_s_ = 0.6 µm, *R*_0_ ≈ 100 kΩ), which decreases the sensitivity of the NTTF*_5_*@fiber sensors. Thus, the sensor with *h*_s_ = 0.8 µm achieved the highest sensitivity.

**Figure 3:**
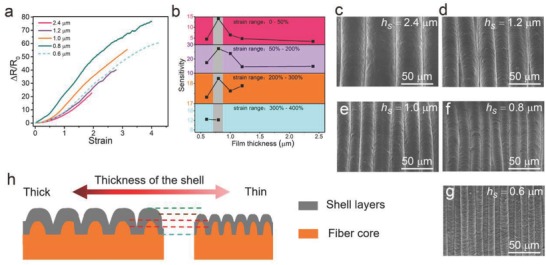
Investigation of the influence of the NTTF thickness on the performance of NTTF*_n_*@fiber sensors. a) Changes in the resistance of NTTF_5_@fiber sensors with different thicknesses (*h*_s_) of the single layer NTTF upon increasing tensile strain. The fabrication strain was 500%, and the number of the layers (*n*) was 5. b) Sensitivities of the NTTF_5_@fiber sensors with different *h*_s_. The values of the NTTF_5_@fiber sensors sensitivities were calculated by piecewise fitting of the curves in (a). c–g) SEM images of the surface morphology of NTTF_5_@fiber sensors with different *h*_s_ at 0% strain. h) Schematic illustration of the section morphology change of NTTF_5_@fiber sensors with different *h*_s_.

Another key factor that affects the sheath layer morphology and performance of the NTTF*_n_*@fiber sensors is the number of the NTTF layers (*n, h* = *n* × *h*_s_). Figure S6a (Supporting Information) shows that NTTF*_n_*@fiber sensors with smaller *n* have a larger strain range (150%, 212.5%, 259%, 317%, 400%, and 450% strain, for *n* = 25, 20, 15, 10, 5, and 3, respectively) due to the constraint on the TPE fiber core provided by the NTTF sheath layers, and a thicker sheath will provide a stronger constraint. The sensitivity (Figure S6b, Supporting Information) and strain range (Figure S8, Supporting Information) change as *n* changes, which is consistent with the results found for sensors with different *h*_s_. SEM images (Figures S7 and S9, Supporting Information) indicate that the lateral contact area decreases as *n* and λ increase. Meanwhile, the contact pathway vanishes and the sensitivity is relatively low with the drastic reduction of the strain range. However, when *n* < 5, the sensitivity also decreases because the drop in conductivity of the NTTF sheath and the overall resistance of the NTTF*_n_*@fiber sensors begins to increase. In this work, the optimal value of *n* is 5, which simultaneously leads to the best sensitivity and largest strain sensing range of the NTTF_5_@fiber sensors.

In spite of the sensitivity, the response time and stability are also important sensing parameters of NTTF*_n_*@fiber sensors. The response time of the sensor was measured by application of quasitransient stretching to the sensor^23^ (Figure S10, Supporting Information). As shown in **Figure** **4**a, the sensor exhibited a response time of only 16 ms, which was much faster than recently reported values for other strain sensors (70–110 ms).^30^, ^36^, ^37^ Figure 4b shows the variation of Δ*R*/*R*_0_ of a strain sensor that underwent 20 000 stretch‐release cycles from 5% to 7% strain. There was no obvious change in the peak values of Δ*R*/*R*_0_, but the baseline slightly decreased. To further test the stability of the strain sensor, another 10 000 stretch‐release cycle tests from 5% to 30% strain was conducted (Figure S11a, Supporting Information). As shown in Figure S11b (Supporting Information), after 2300 repeated cycles, the sensing ability tended to be stable and the peak and baseline did not change. Consequently, it could be concluded that the NTTF sheath layer was firmly combined with the fiber core and the buckled structure was stable. Moreover, with a smaller film thickness (≈0.8 × 5 µm), it will take less time to balance the repositioning of the buckled structure on the NTTF sheath while the sensor is under strain, resulting in a faster response time.

**Figure 4:**
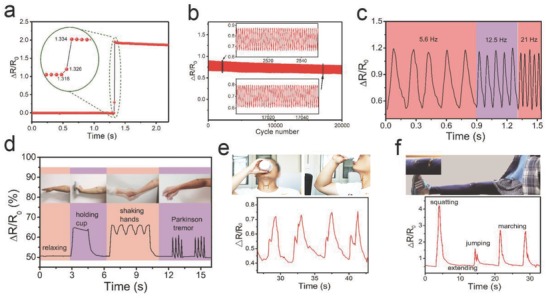
Sensing performance of NTTF*_n_*@fiber sensors and real‐time muscular movement and human motion detection. a) Real‐time response of a sensor upon application of a quasitransient step strain from 0% to 5%. Inset: Enlarged Figure showing the response time. b) Resistance change‐time plot for more than 20 000 stretch/release cycles at 0.6 s for each cycle with an applied strain of 2%. The testing apparatus is shown in Figure S11a (Supporting information). c) Real‐time variation in the relative resistance under repetitive stretching from ε = 5% to ε = 20% at different frequencies. d–f) Response to motions of d) arm muscle with different gestures, e) the throat when drinking, and f) the knee joint. The NTTF*_n_*@fiber sensor used was encapsulated by spray coating a thin (≈50 nm) TPE film on a relaxed fiber sensor, which did not have a significant effect on the sensors performance. Parameters of the tested strain sensor: ε_pre_ = 500%, *n* = 5, and *h*_s_ = 800 nm.

In modern medical practice, continuous human motion monitoring provides a lot of useful information for disease prevention and medical diagnosis.^38^ As most human actions are dynamic processes with certain frequencies, the response to the dynamic strain of the fiber sensor was investigated by measuring the relative resistance change under repetitive stretching from ε = 0% to ε = 20% at different frequencies (0–21 Hz) and repetitive stretching from ε = 20% to ε = 75% at a constant frequency (0.2 Hz), as shown in Figure 4c; and Figure S12 (Supporting Information), respectively. The sensor exhibited high repeatability and small hysteresis for real‐time dynamic detection, making it applicable to the detection of human activities with different frequencies. Considering that fiber sensors may contact the skin or organs of humans, we encapsulated the NTTF_5_@fiber sensor by spray coating a thin (≈50 nm) TPE film onto it in a relaxed state; this film did not have a significant effect on the sensor performance (Figure S13, Supporting Information). As a demonstration, a flexible bracelet composed of the fiber strain sensor was pasted onto a person's arm to monitor subtle arm muscle motion (Figure 4d). The characteristic waveform of sensor resistance changes were able to clearly reflect different arm muscle motions, such as extending, holding cup, shaking hands, and simulating a Parkinson's tremor. Then, the fiber strain sensor was attached to a person's throat to detect related muscle movements during swallowing, and subtle movements were reflected by the different values of the relative resistance change (Figure 4c). These experiments demonstrated the ability of the fiber strain sensor to monitor subtle deformations in real time (Video S1, Supporting Information).

On the other hand, the large workable strain range allowed the sensor to recognize large deformations of the human body. For example, measurements were successfully conducted on cervical vertebra during bowing for long times and quick nodding (Figure S14a and Video S2, Supporting Information), which can provide guidance for preventing the occurrence of cervical spondylosis and spine injuries. When the sensor was fixed to the knee‐joint, motion, such as squatting, extending, jumping, and marching, was able to be monitored in real‐time (Figure 4f). The fiber strain sensor was also fixed on the elbow joint to detect bending of the forearm. When the extended forearm bent to a certain angle, the relative resistance changes of the strain sensor rose to a corresponding value rapidly (Figure S14b and Video S1, Supporting Information).

The tendon is a continuation of an internal and external muscle; the tendon works as a transmission structure for dexterous movements.^39^ Tendon injury has a large impact on human articular function, while tendon rupture is a very common injury, particularly for athletes. In recent years, tendon rupture rehabilitation has received great attention, and development of an effective rehabilitation assessment method is of great importance for early recovery exercise and repair. In this work, a series of animal experiments was conducted by attaching the sensor as an implantable device to the tendon of a lab rat for real‐time quantitative assessment of tendon rehabilitation. The ultrastretchable strain sensor with a diameter of 200 µm was encapsulated by a TPE thin film (≈50 nm) and fixed to the hamstring of a lab rat with an injured leg (**Figure** **5**a). The angle of the tibia and metatarsus in the relaxed state was noted as θ_1_ (Figure 5b) and that under the stretching state was noted as θ_2_. θ_3_ ( =θ_2_ – θ_1_) represented the leg stretching exercise level of the rat, which was driven by the self‐custom force. Figure 5c presents real‐time Δ*R/R*_0_ *–t* curves of the strain sensor when the rat was driven to perform cyclic stretching exercises at two different levels (θ_3_ = 45° and 90°). The strain sensor showed a stable and reproducible performance and was able to detect angle alternations (Video S3, Supporting Information). Figure S15 (Supporting Information) shows the correlations of the strain to force and force to a relative resistance change, which endow the strain sensor with the ability to perform real‐time monitoring of the loaded force from the muscle to the ankle. This ability may play a key role in rehabilitation assessment for guiding rehabilitation training.

**Figure 5:**
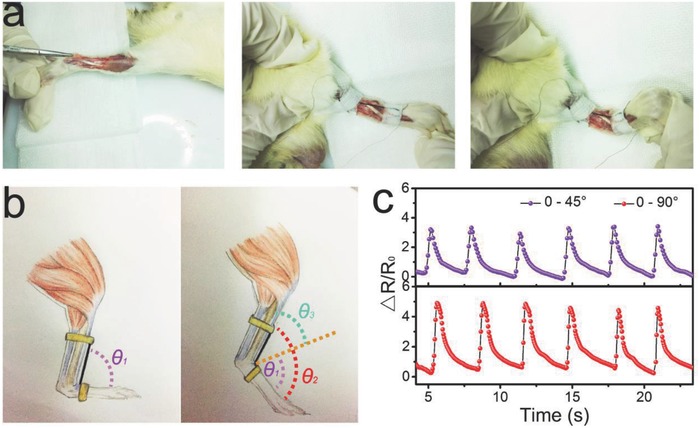
Quantitative assessment of tendon rehabilitation using a fiber strain sensor. a) Optical images showing the process of fixing the strain sensor to the hamstring of a lab rat with an injured leg. Left: The hamstring of a lab rat with an injured leg without sensors. Middle: The hamstring of a lab rat with an injured leg with fiber sensors and θ_3_ = 90°. Right: θ_3_ = 45°. θ_3_ is defined as in (b). The used NTTF_5_@fiber sensor was encapsulated by spray coating a thin (≈50 nm) TPE film on a relaxed fiber sensor, which did not have significant effect on the sensors performance. b) Schematic illustration of a strain sensor on the hamstring of a rat leg, and the angle of the tibia and metatarsus under the relaxed state was denoted as θ_1_ and that the under stretching state was denoted θ_2_. θ_3_ (θ_3_ = θ_2_ – θ_1_) was defined as the angle of the ankle joint. c) Relative resistance change of the strain sensor as a function of time when the rat was doing cyclic leg stretching exercises with different levels.

In summary, an ultrastretchable buckled sheath–core NTTF*_n_*@fiber sensor was developed by a prestretching‐wrapping‐releasing strategy. Compared with conventional strain sensors, the sheath–core fiber strain sensor displays excellent stretchability (up to 1135%), high sensitivity in whole strain range (GF of 21.3 for 0–150% strain range and 34.22 for 200–1135% strain range), fast response time, and good reproducibility and stability. This high performance was achieved by employing a multilayer buckled structure to the strain‐sensitive NTTF sheath. Moreover, the sensors can be used for real‐time detection of various human motions and as potential implantable devices to quantitatively assess tendon rehabilitation. We believe that this novel ultrastretchable strain sensor has great potential in healthcare and sports.

## Experimental Section

*Materials*: MWCNTs with an average length of 0.5–2 µm and average diameter of 10–20 nm (purity > 95 wt%) were purchased from Nanjing XFNANO Materials Tech Co., Ltd., China. Cyclohexane and ethanol of analytical grade were purchased from Sinopharm Chemical Reagent Co., Ltd., China. Polystyrene‐*block*‐polyisoprene‐*block*‐polystyrene (thermoplastic elastomer, TPE) was purchased from Gainshine incorporation, China. All materials were used as received.

*Ink and Fabrication of a MWCNT/TPE Composite Film (NTTF)*: Sixty milligrams of MWCNTs were dispersed in 200 mL of cyclohexane by strong sonication (300 W) for 1.5 h using an ultrasonic cell disruptor (BILON 92‐II, Shanghai Bilon Co., Ltd.), followed by dissolving different amounts of a TPE elastomer via magnetic stirring for 10 min and an additional 1.5 h of sonication (300 W) to form a suspension (ink). After another hour of sonication, the suspension was centrifuged at a centrifugal force of 9600 *g* for 10 min to remove solid residues. The prepared supernatant was then spray coated on a clean silica glass slide to form a thin MWCNT/TPE composite film. The air pressure for spraying was kept at 20 psi, the distance between the spray‐gun and glass substrates was ≈10 cm, and the spraying speed was 0.5 cm s^−1^. Thereafter, the coated glass substrates were immersed in absolute ethanol for 2 min and dried in air for at least 5 min. NTTFs with different thicknesses were obtained by controlling the amount of spray coating, and the relationship between the spray quantity and the thickness was consistent with the equation: *h*_s_ = 5 × *V*, where the unit of *h*_s_ and *V* are µm and mL cm^−2^.

*Sensor Preparation*: Ultrastretchable fiber sensors were prepared by wrapping a MWCNT/TPE composite film around a TPE fiber core. TPE fiber cores with different diameters were processed from purchased as TPE particles and assembled via twin‐screw extrusion (Kesun Plastic Equipment Co., Ltd., China). The process for fabricating the sensor is illustrated in Figure 1A. First, a TPE fiber was fixed on two motors that synchronously rotated, and then, the TPE fiber was stretched and the strain was kept 1600%. The silica glass slide with the MWCNT/TPE composite film was placed on a translation stage. Ethanol was dropped onto the surface of the film to make it easy to wrap. The two motors synchronously rotated the prestretched TPE fiber and the composite film was brought into contact with the rotating rubber fiber slowly, allowing it to be wrapped around the fiber, and the number of turns of the rubber fiber was denoted as the number of NTTFs. After drying in air, the MWCNT/TPE composite film wrapped fiber sensor with a buckled surface structure was obtained after the prestrain was released.

*Characterizations and Sensing Experiments*: A digital camera (Canon EOS 70D) was used to take photos and record videos in this paper. Tensile tests were conducted using an Instron mechanical tester (Model 5969). The sheet resistance of the MWCNT/TPE composite films was observed using a multifunction digital four‐probe tester (JG, ST‐2258C). SEM was performed for micromorphology observation with a Hitachi S‐4800 cold field emission SEM at an accelerating voltage of 5 kV. A stepper machine (BeiJing Optical Century Instrument Co., Ltd., China) was used to stretch the fiber strain sensor with one end fixed and the other end elongated linearly at a constant speed. Resistance measurements were carried out by connecting the two ends of the fiber strain sensors to a digital source meter (Keithley 2602A), with two copper wires as the electrodes to record the real‐time electric current (*I*) of the sensor under a constant voltage (*V*_0_) of 2 V, and the real‐time resistance (*R*) was calculated as *R* = *V*_0_/*I*. Experiments with human subjects were obtained from the individuals with signed consent. This work is not about living individuals and does not include collection of individually identifiable private information, therefore, the IRB (Institutional Review Board) approval was not a prerequisite.

*Quantitative Assessment of Tendon Rehabilitation*: Male Sprague‐Dawley 12‐week‐old rats (weight: 220–250 g) were provided by Chongqing Daping hospital. Before the experiment, the lab rats were fasted for 12 h and provided water ad libitum. Pentobarbital was injected into the abdomen at a dose of 30 mg kg^−1^ to anesthetize the rats, and the room temperature was maintained at 22.5 °C. In a sterile environment, the epidermis was cut along the front of the tibia of rats. Then, one end of the fiber strain sensor was fixed to the tibia and the other end was fixed to the metatarsal bone, and two Cu wire electrodes were connected to both ends of the fiber sensor. A digital source meter was used to measure the real‐time electric current during the test, allowing real‐time quantitative assessment of tendon rehabilitation. The animal experiments were approved by the Laboratory Animal Welfare and Ethics Committee of the Third Military Medical University with a certification (the accreditation number of the Laboratory Animal Welfare and Ethics Committee: SYXK 20170002).

## Conflict of Interest

The authors declare no conflict of interest.

## Supporting information

Supplementary

Supplementary

Supplementary

Supplementary
