# Supplementary material for: Ultrastretchable Fiber Sensor with High Sensitivity in Whole Workable Range for Wearable Electronics and Implantable Medicine
Source: Adv Sci (Weinh). 2018 Jul 23;5(9):1800558. doi: 10.1002/advs.201800558 (PMC6145303; doi:10.1002/advs.201800558)
Supplement: Supplementary file 1 — Supplementary [file ADVS-5-1800558-s002.pdf]

Copyright WILEY-VCH Verlag GmbH & Co. KGaA, 69469 Weinheim, Germany, 2018.

Supporting Information

for *Adv. Sci.,* DOI: 10.1002/advs.201800558

Ultrastretchable Fiber Sensor with High Sensitivity in Whole Workable Range for Wearable Electronics and Implantable Medicine

*Lianhui Li, Hongyi Xiang, Yan Xiong, Hui Zhao, Yuanyuan Bai, Shuqi Wang, Fuqin Sun, Mingming Hao, Lin Liu, Tie Li, Zhenhuan Peng, Jiaqiang Xu, and Ting Zhang**

Copyright WILEY-VCH Verlag GmbH & Co. KGaA, 69469 Weinheim, Germany, 2016.

Supporting Information

Title **Ultrastretchable Fiber Sensor with High Sensitivity in Whole Workable Range for Wearable Electronics and Implantable Medicine**

*Lianhui Li, Hongyi Xiang, Yan Xiong, Hui Zhao, Fuqin Sun, Yuanyuan Bai, Shuqi Wang, Mingming Hao, Lin Liu, Tie Li, Zhenhuan Peng, Jiaqiang Xu, Ting Zhang**

Supplementary Figures and Figure Captions

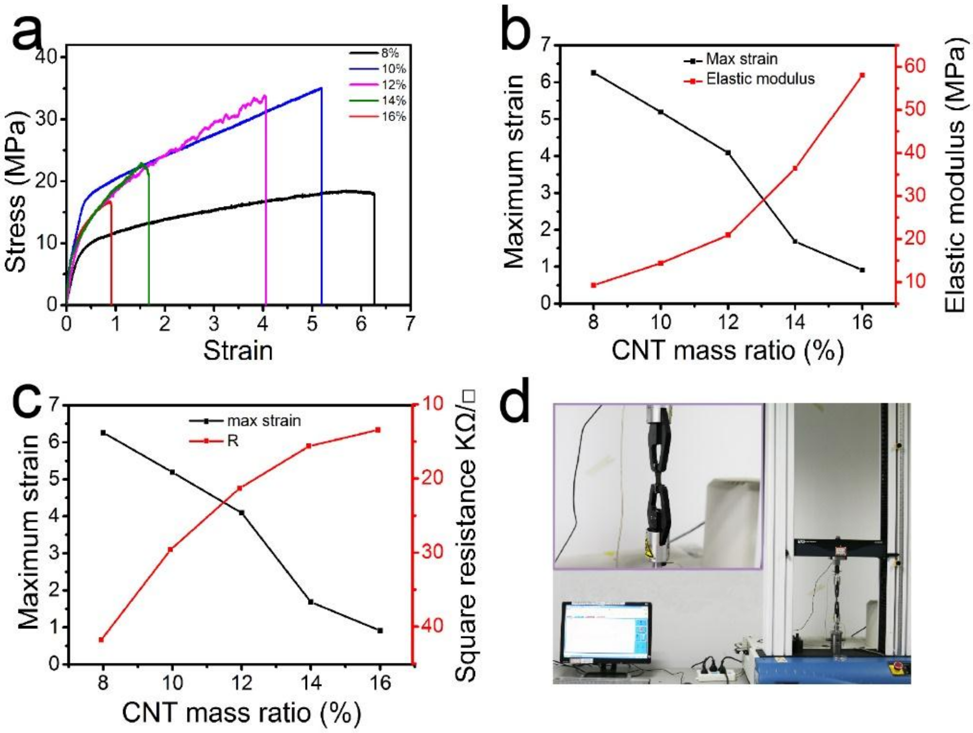

Figure S1 a) Stress-strain curves for different MWCNT/TPE composite films (NTTF) with various MWCNT weigh ratios. The MWCNTs content is calculated as the value of $M_{MWCNT}/(M_{MWCNT}+M_{TPE})$, where M is the weight of MWCNTs or TPE. The experiment was set to stop at breaking of the films with a constant stretching speed of 50% min^{-1}. b) Plot of the maximum strain and elastic modulus as a function of the MWCNTs mass ratio. c) Maximum strain and square resistance as a function of the MWCNTs mass ratio. d) Optical photograph of an Instron mechanical tester (Model 5969). The inset shows a photograph of the tensile test with a stretching machine. To quantify the mechanical properties of strain sensors with different wrapping layers, tensile tests were conducted using the Instron mechanical tester. As shown in d, the strain sensor was fixed between two air jigs of the mechanical tester and then stretched uniaxially at a constant speed of 10 mm· min^{-1} until the wrapping layers ruptured.

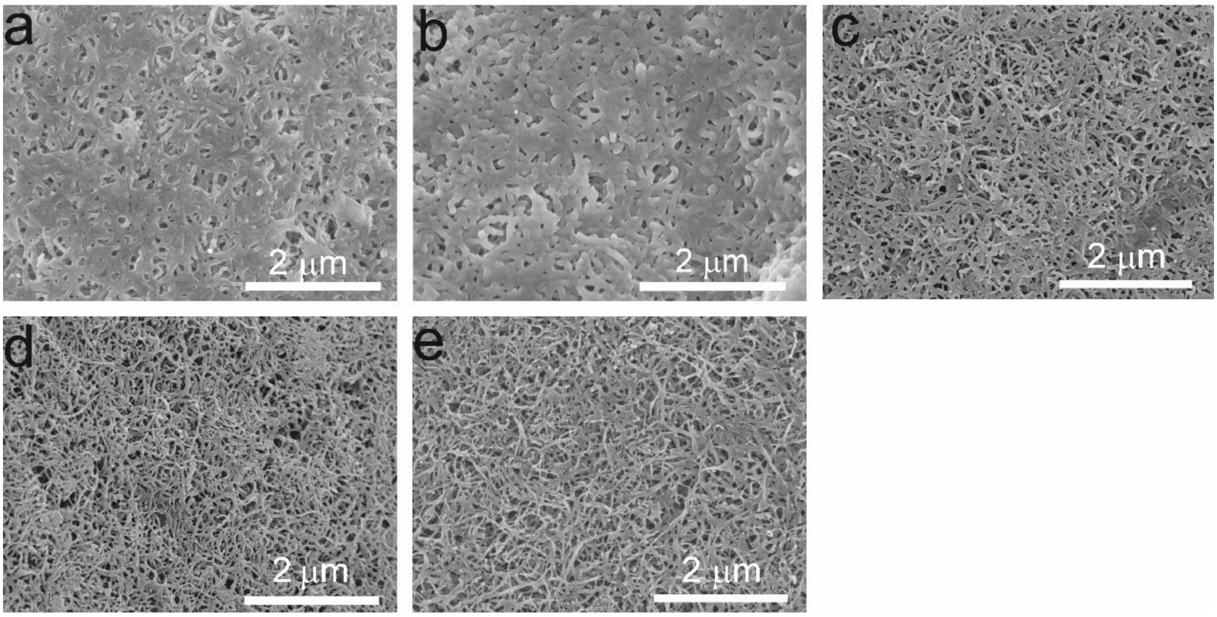

Figure S2 a-e) SEM images of NTTFs with different MWCNT weight ratio (a-e: 8, 10, 12, 14 and 16 wt%). All the films were soaked in ethanol for 2 min and dried by nitrogen gun.

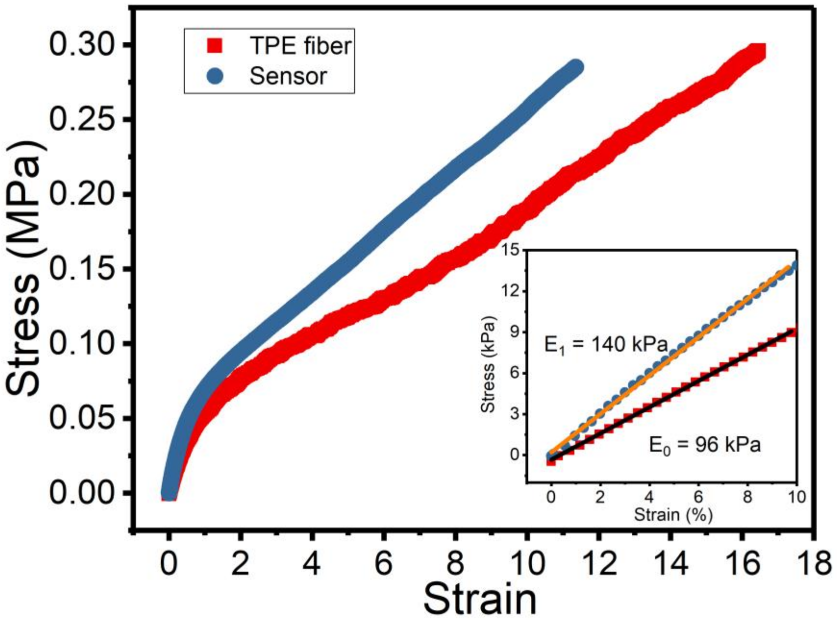

Figure S3 Stress-strain curves for TPE fiber and $NTTF_5$@fiber sensor. The fabrication strain of the $NTTF_5$@fiber sensor was 1600%, and the thickness of the NTTF was 800 nm.

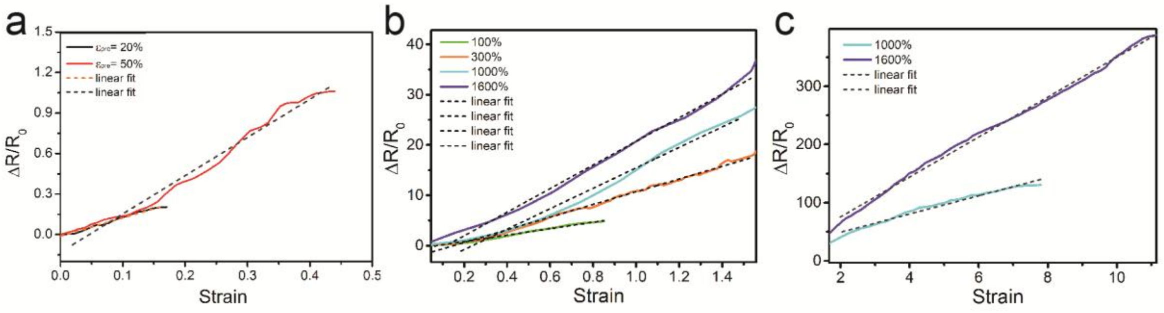

Figure S4 a-c Resistance change as a function of strain for NTTF$_5$@fiber with different ε_{pre}. The dotted lines are the linear fittings of different curves.

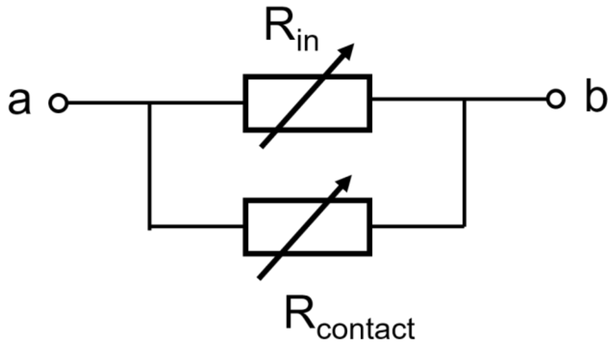

Figure S5 Two resistors (R_{in} and R_{contact}) that are in parallel show the resistor network model for conductance along the NTTF$_n$@fiber.

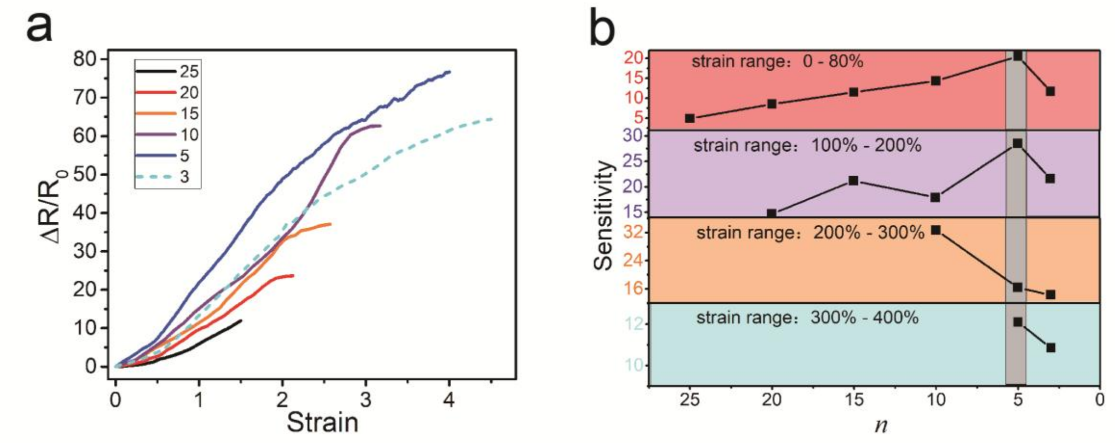

Figure S6 a) Change in resistance of the NTTF$_n$@fiber sensors with different numbers (*n*) of MWCNT/TPE composite films (NTTF) on increasing tensile strain. The fabrication strain was 500% and the thickness of the NTTF was 800 nm. b) The sensitivities of the NTTF$_n$@fiber sensors with different *n*. The values of the NTTF$_n$@fiber sensors sensitivities were calculated by piecewise fitting of curves in (a).

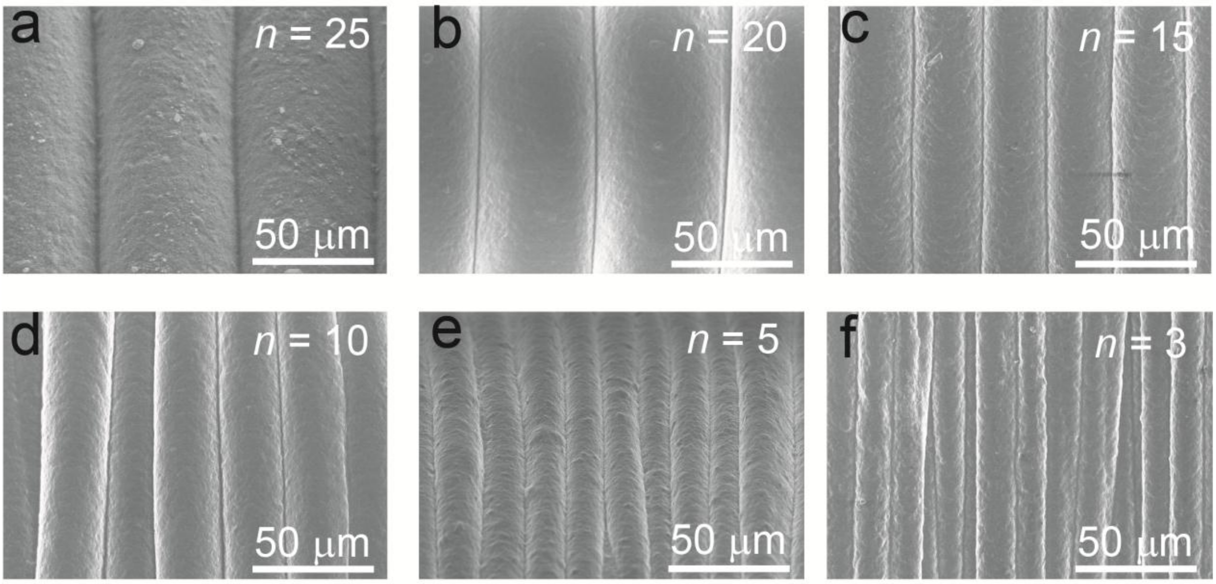

Figure S7 a-f) SEM images of the surface morphology of the NTTF$_n$@fiber sensors with different *n* at 0% strain. The fabrication strain (ε_{pre}) was 500% and the thickness of the NTTF was 800 nm.

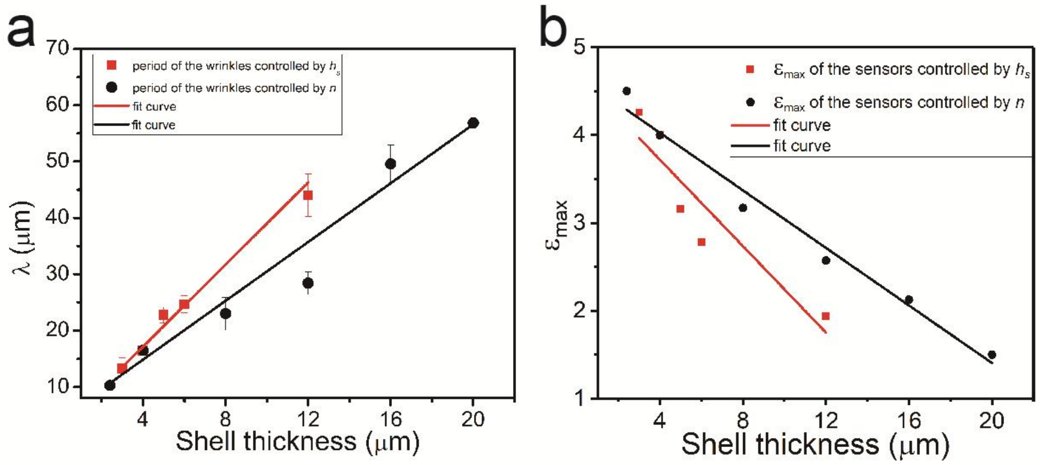

Figure S8 a) period (λ) of the buckles on the sheath layer as a functionof the shell thickness (h). $h = n*h_s$. The fabrication strain (ε_{pre})of the sensors are 500%. The red square is the data of the NTTF$_n$@fiber sensors that only had different thickness (h_s) of single layer NTTF. The black dot is the data of the NTTF$_n$@fiber sensors that only had different nubmers (n) of NTTFs. The corresponding solid lines are the linear fitting curves. b) the maximum strain range (ε_{max}) as a functionof the shell thickness (h).

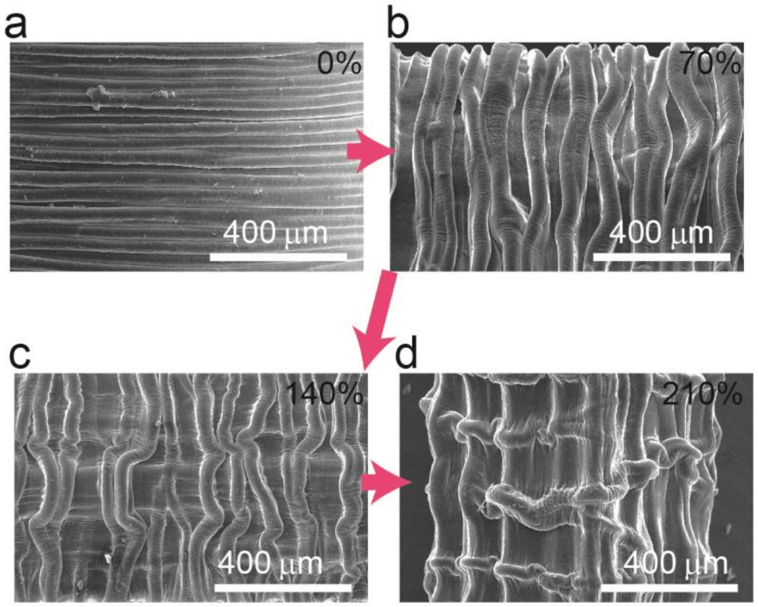

Figure S9 SEM images of the surface morphology of the $NTTF_{20}$@fiber sensor. The fabrication of the strain sensor was 500% and the thickness of the NTTF was 800 nm.

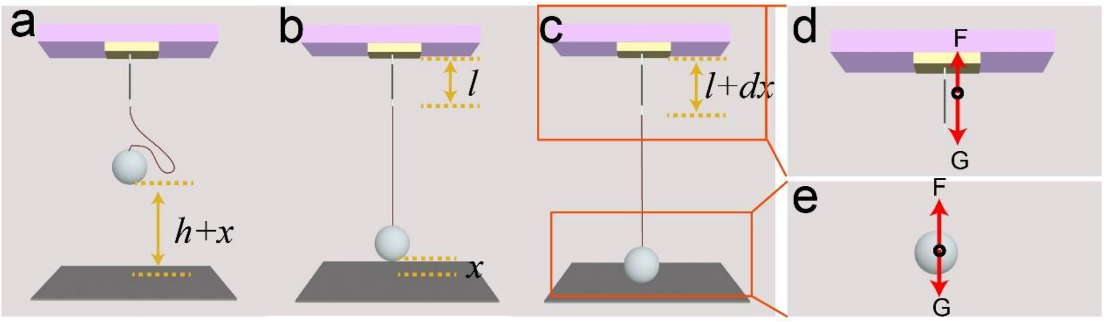

Figure S10. Schematics showing the measurement procedure of the response time of the ultrastrechable fiber strain sensor. a) Initial state. b) Critical state. c) Tensile state. d-e) Schematic illustrations showing the forces acting on the TPE fiber and the iron ball.

Fiber strain sensor with a length (l) of 40 mm was connected to the iron ball (500 g) using two polyester threads. Bottom of the iron ball and ground surface below it was both stuck with a thin layer of double-sided adhesive (~0.05 mm). At the initial state, the sensor and one of the connecting thread was relaxed, while the other was tensioned. The iron ball was in the position right under the fiber sensor with a height of $h+x$. In the following step, by cutting off the tensioned thread, the iron ball dropped vertically due to its own gravity. At a critical state that the remaining connecting thread was just tensioned, and the TPE fiber was still relaxed, suppose that the iron ball had fallen by a height of h, so its real-time height at the critical state was x. Velocity of the ball could be calculated as $v_0 = \sqrt{2gh}$ from the energy conservation law $\left(mgh = \frac{1}{2}m{v_0}^2\right)$, where $9.8\ m/s^2$ was gravitational acceleration. After that, as the iron ball continued falling down by a height of dx, the fiber sensor would be stretched gradually to a length a dx. During the process, the TPE fiber was under action of a resilience force caused by stretching (F) and a pulling force originating from gravity of the connecting iron ball (G), while the iron ball was under action of pulling force from the TPE fiber (equal to F) and its own gravity. According to Newton's second law, acceleration of the iron ball would be $a = \frac{G-F}{m}$, where $G = mg = 4.9\ N$ and F could be measured afterwards. When dx was tiny, a tiny time of dt would be lasted, and velocity of the iron ball would be $v = v_0 + a \cdot dt = v_0 + \left(\frac{G-F}{m}\right) \cdot dt$. As the iron ball fell down on the ground and stuck by the adhesive on it, tensile strain of the sensor at that time would be $\varepsilon = \frac{x}{l}$, where x could be

calculated as $x=\int dx=\int v\cdot dt=\int\left(v_0+\left(\frac{G-F}{m}\right)\cdot dt\right)\cdot dt$. In our measurement, *h* and *x* was set to be 100 mm and 2 mm respectively, and v_0, ε was calculated to be 1.4 m/s, 5% respectively. It could be seen from Fig. 2a that the resilience force of TPE fiber caused by stretching of $\varepsilon<5\%$ was smaller than 0.023 N, and so it could be concluded that $G-F>0$, and $x=\int\left(v_0+\left(\frac{G-F}{m}\right)\cdot dt\right)\cdot dt>\int v_0\cdot dt=v_0t$, ie. $t<x/v_0=1.4\ s$. Therefore, the stretching procedure of the sensor from the relaxed state of ε=0% to the tensile state of ε=5% could be seen as a quasi-transient process, and by recording time response of the sensor, its response time could be determined from the $\Delta R/R_0-t$ curve, as shown in Fig. 3b.

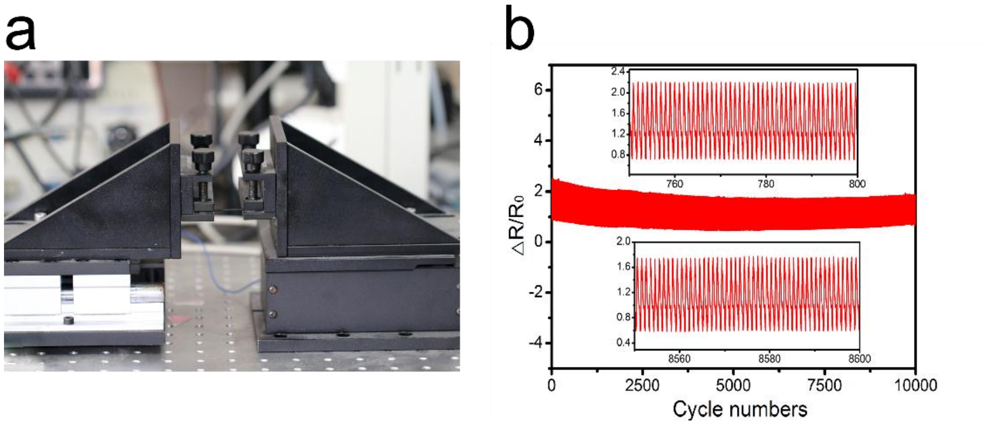

Figure S11 a) Optical photograph of stepper motor used for the stability test. b) Resistance change-time plot for more than 10000 stretch/release cycles, at 4.6 s for each cycle, with applied strain range from 5% to 30%.

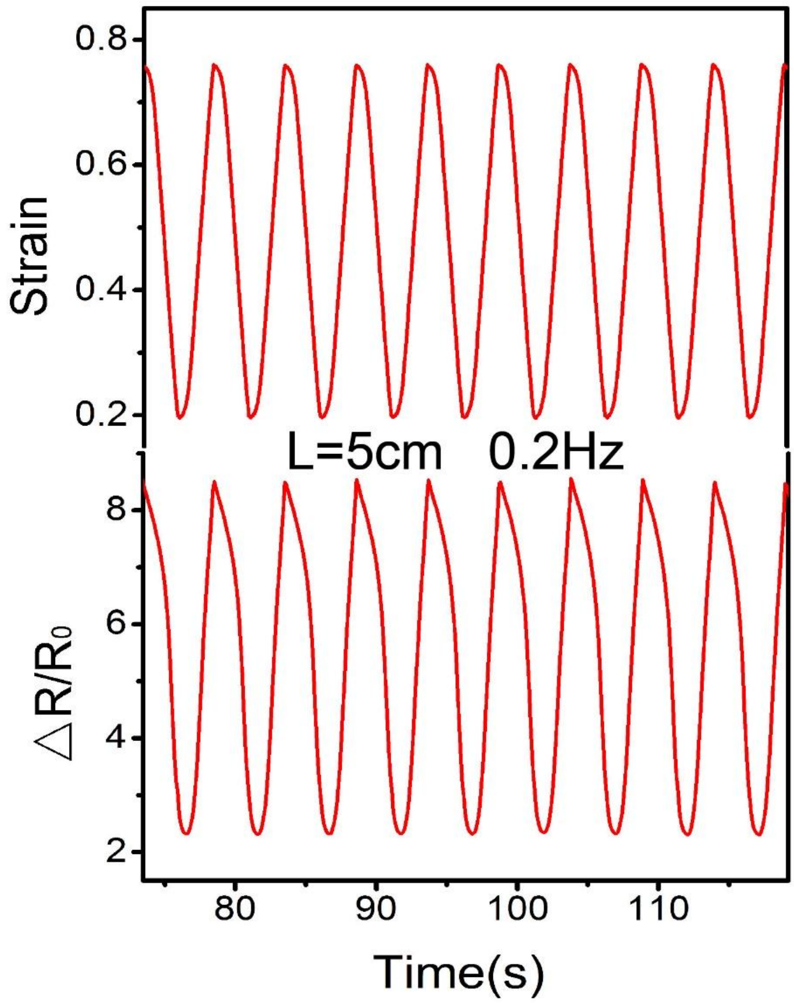

Figure S12 Response of the $NTTF_5$@fiber sensor unit to applied strain (50%) with a frequency of 0.2 Hz. The fabrication (ε_{pre}) was 1600% and the thickness of NTTF was 800 nm.

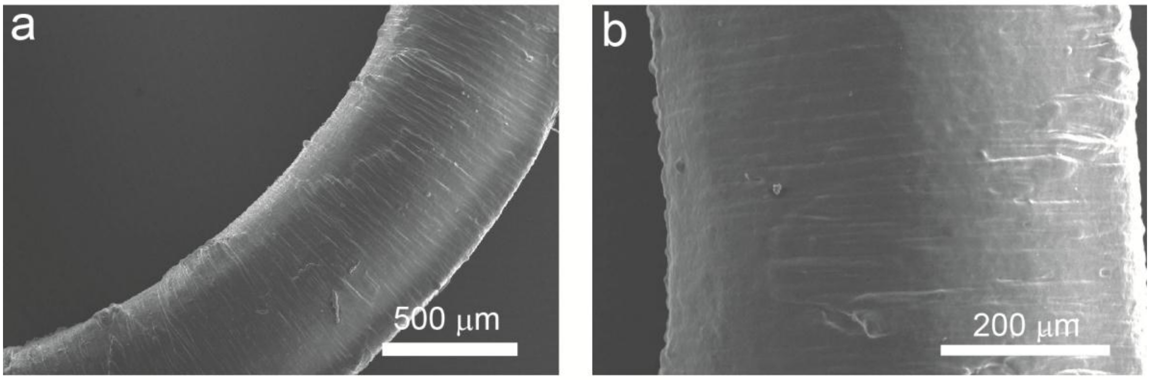

Figure S13 SEM images of the NTTF$_n$@fiber sensor encapsulated by the TPE film. The used NTTF$_5$@fiber sensor was encapsulated by spray coating a thin (~10 nm) TPE film on a relaxed fiber sensor, which did not have significant effect on the sensors performance.

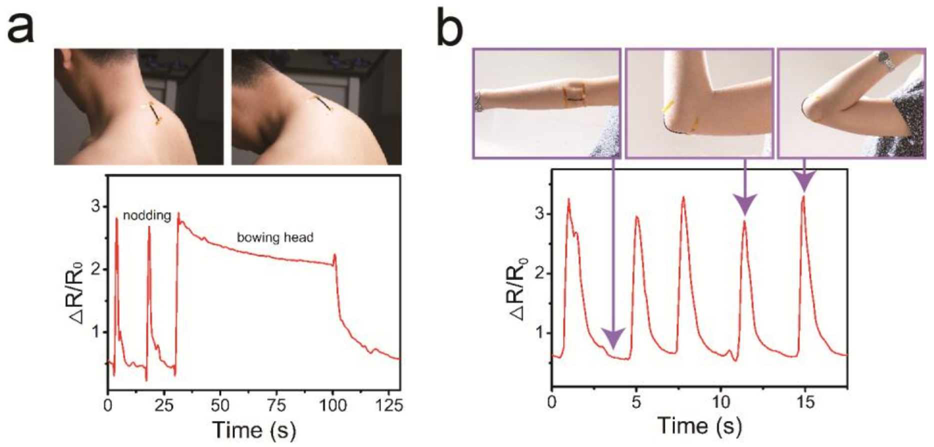

Figure S14 Response to motions of a) cervical vertebrae, b) elbow joint. Insets in each panel show the photographs of the strain sensors at different conditions.

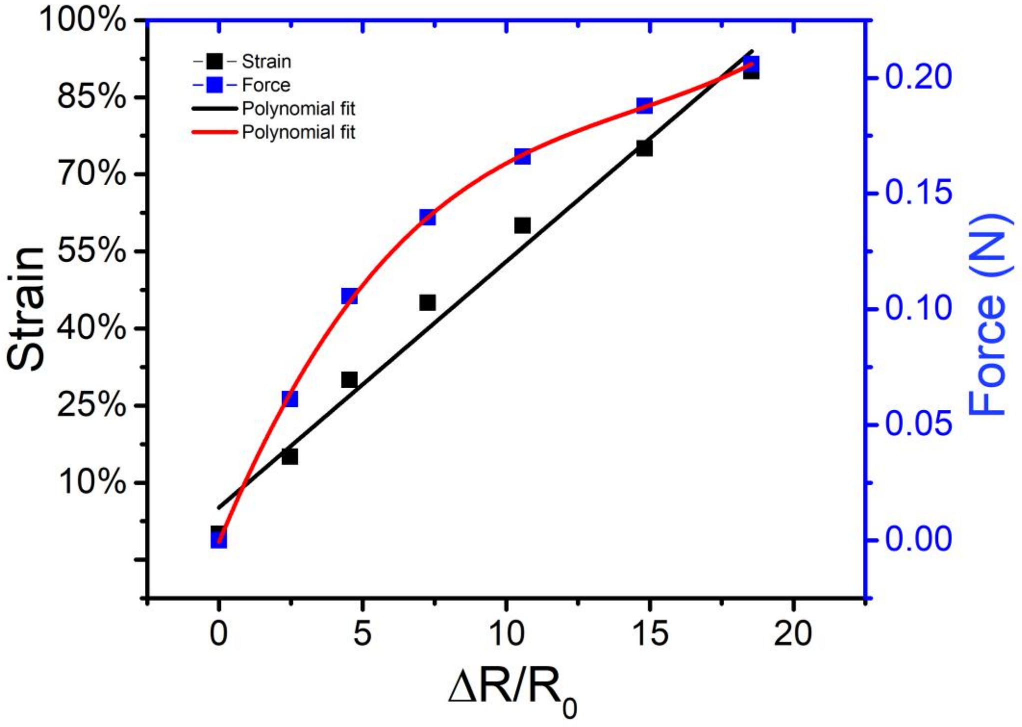

Figure S15 Strain and force change as a function of relative resistance change. The fabrication (ε_{pre}) of the NTTF$_n$@fiber strain sensor was 1600%, the thickness of NTTF was 800 nm and n = 5.

Supplementary Movie Captions

Movie S1. Real-time monitoring of subtle and large deformations. A flexible bracelet made from the ultrastretchable fiber strain sensor was worn on the arm; then, a digital source meter (Keithley 2602A) was used to measure the real-time electric current change of the strain sensor. Initially, the hand was slowly and repeatedly clenched and relaxed, and its electric current change showed a gradual and periodic synchronous increase and decrease, realizing real-time detection of subtle deformation of the arm muscles. Later, a fiber strain sensor was fixed on the index finger to monitor large-strain actions.

Movie S2. Dynamic and static motion monitoring. A fiber strain sensor was fixed on the inner side of an index finger to detect dynamic and static motions. The index finger was rapidly bent to three different angles, held for 3 s at each angle, and then relaxed. A digital source meter (Keithley 2602A) was used to measure the real-time electric current.

Movie S3. Real-time quantitative assessment for tendon rehabilitation of lab rats. Male Sprague-Dawley rats aged 12 weeks (weight: 220 g ~ 250 g) were provided by Chongqing Daping hospital. Before the experiment, lab rats were fasted for 12 h, with water provided ad libitum. Pentobarbital was injected into the abdomen at a dose of 30 mg kg^{-1} to anesthetize the rats, and the room temperature was maintained at 22.5 ℃. In a sterile environment, the epidermis was cut along the front of the tibia of rats. Then, one end of the encapsulated fiber strain sensor was fixed on the tibia and the other end was fixed on the metatarsal bone, and two Cu wire electrodes were connected on both ends of the fiber sensor. A digital source meter was used to measure the real-time electric current during the test, realizing real-time quantitative assessment of tendon rehabilitation.
